# Gangliosides Block *Aggregatibacter Actinomycetemcomitans* Leukotoxin (LtxA)-Mediated Hemolysis

**DOI:** 10.3390/toxins2122824

**Published:** 2010-12-14

**Authors:** Michael S. Forman, Jason B. Nishikubo, Rebecca K. Han, Amy Le, Nataliya V. Balashova, Scott C. Kachlany

**Affiliations:** Department of Oral Biology, New Jersey Dental School, University of Medicine and Dentistry of New Jersey, 185 South Orange Avenue, Newark, NJ 07103, USA; Email: mforman@gwmail.gwu.edu (M.S.F.); nishikjb@umdnj.edu (J.B.N.); rebeccahan88@yahoo.com (R.K.H.); amethist1983@yahoo.com (A.L.); balashnv@umdnj.edu (N.V.B.)

**Keywords:** erythrocytes, toxin, periodontal disease, endocarditis, RTX toxin

## Abstract

*Aggregatibacter actinomycetemcomitans* is an oral pathogen and etiologic agent of localized aggressive periodontitis. The bacterium is also a cardiovascular pathogen causing infective endocarditis. *A. actinomycetemcomitans* produces leukotoxin (LtxA), an important virulence factor that targets white blood cells (WBCs) and plays a role in immune evasion during disease. The functional receptor for LtxA on WBCs is leukocyte function antigen-1 (LFA-1), a β-2 integrin that is modified with N-linked carbohydrates. Interaction between toxin and receptor leads to cell death. We recently discovered that LtxA can also lyse red blood cells (RBCs) and hemolysis may be important for pathogenesis of *A. actinomycetemcomitans*. In this study, we further investigated how LtxA might recognize and lyse RBCs. We found that, in contrast to a related toxin, *E. coli* α-hemolysin, LtxA does not recognize glycophorin on RBCs. However, gangliosides were able to completely block LtxA-mediated hemolysis. Furthermore, LtxA did not show a preference for any individual ganglioside. LtxA also bound to ganglioside-rich C6 rat glioma cells, but did not kill them. Interaction between LtxA and C6 cells could be blocked by gangliosides with no apparent specificity. Gangliosides were only partially effective at preventing LtxA-mediated cytotoxicity of WBCs, and the effect was only observed when a high ratio of ganglioside:LtxA was used over a short incubation period. Based on the results presented here, we suggest that because of the similarity between N-linked sugars on LFA-1 and the structures of gangliosides, LtxA may have acquired the ability to lyse RBCs.

## 1. Introduction

*Aggregatibacter actinomycetemcomitans* is a gram negative oral bacterium that can cause localized aggressive periodontitis in adolescents (LAP) [1,2,3]. The bacterium is also part of the normal oral flora in many healthy individuals [4,5]. The disease occurs predominantly in African Americans and approximately 70,000 adolescents develop the disease in the U.S. per year [6]. LAP is a destructive form of periodontitis that affects the central incisors and first molars, and LAP results in the rapid loss of bone and periodontal ligament surrounding the teeth. When untreated, patients who suffer from LAP often lose their affected teeth. In addition to being an important oral pathogen, *A. actinomycetemcomitans* is part of the HACEK group of bacteria (*Haemophilus spp.*, *A. actinomycetemcomitans*, *Cardiobacterium hominis*, *Eikenella corrodens*, and *Kingella kingae*) implicated in infective endocarditis (IE) [7,8] and *A. actinomycetemcomitans* is reported to be the HACEK organism involved most often in IE [9].

*A. actinomycetemcomitans* produces numerous virulence factors including leukotoxin (LtxA), which targets human and Old World primate white blood cells (WBC; reviewed recently in [10]). LtxA is an ~114 kDa secreted protein [11] and is a member of the repeats in toxin (RTX) family of bacterial toxins. Other RTX toxins include *Escherichia coli* HlyA, *Bordetella pertussis* CyaA, *Mannheimia haemolytica* LktA, *Actinobacillus pleuropneumoniae* Apx toxins, and *Vibrio cholerae* RtxA [12]. Like HlyA and CyaA, LtxA has been shown to be modified with fatty acids linked to internal lysine residues and this modification is required for activity [13]. LtxA is believed to play an important role in immune evasion by selectively depleting host WBCs that target the bacterium. 

The WBC receptor for LtxA is leukocyte function antigen-1 (LFA-1) [14]. LFA-1 is a β2-integrin composed of the subunits CD11a and CD18. These molecules are modified with N-linked oligosaccharides [15] and can exist in at least two different functional states [16,17]. After binding to LFA-1, LtxA causes a signaling cascade that results in apoptosis when used at low concentrations. While the mechanism has not been completely deciphered, LtxA appears to intoxicate cells via an apoptotic pathway that involves the mitochondria [18]. However, at high concentrations (greater than 5 μg/mL), LtxA kills cells very rapidly by necrosis. We recently reported that LtxA preferentially kills cells with activated LFA-1 [19]. Thus, rather than killing all WBCs non-specifically, *A. actinomycetemcomitans* LtxA targets immune cells that are most immunocompetent and effective at eliminating microbial pathogens. 

Several years ago, we discovered that LtxA could also lyse red blood cells (RBCs) from a variety of species including human, sheep, and horse [20]. RBCs do not express LFA-1, and so it is not known how LtxA interacts with these cells. The concentration of LtxA required for hemolysis of RBCs was higher than that needed for killing of WBCs, signifying a higher affinity receptor on WBCs or a greater number of receptors [20]. We also found that free iron repressed the secretion of LtxA from bacteria, which suggested a role for hemolysis in iron acquisition [21]. Because there is no available free iron in the host, pathogens have evolved numerous mechanisms of releasing and sequestering iron often in the form of heme. Coupling LtxA-mediated hemolysis to other iron acquisition mechanisms may be important for survival and persistence of *A. actinomycetemcomitans* in the host. 

Other RTX leukotoxins have also been shown to possess hemolytic activity, including *M. haemolytica* LktA [22]. Cortajarena *et al.* [23] previously reported that another RTX toxin, *E. coli* α-hemolysin, recognizes glycophorin on the surface of RBCs, which results in hemolysis. Because of our recent observation that LtxA has hemolytic properties, we further investigated a potential RBC surface component that is recognized by the toxin. We report here that LtxA does not use glycophorin as a receptor, but instead is blocked by and possibly interacts with gangliosides, which are lipid-sugar molecules expressed on the surfaces of cells.

## 2. Materials and Methods

### 2.1. Cells

HL-60 (CCL-240), THP-1 (TIB-202), K562 (CCL-243) (human leukemia cell lines) and C6 rat glioma cells (CCL-107) were obtained from ATCC (Manassas, VA). HL-60, THP-1, and K562 cells were maintained in RPMI 1640 medium with 10% fetal bovine serum (Life Technologies, Carlsbad, CA) at 37 °C, 5% CO_2_. Cells were grown for several days until cell concentration reached approximately 1.0 × 10^6^ cells/mL. Rat glioma C6 cells were grown in Hams Nutrient Mixture F-12 (Fisher Scientific, Pittsburgh, PA) with 15% horse serum, 2.5% fetal bovine serum. *A. actinomycetemcomitans* strain NJ4500 was used for LtxA purification and was grown as described [24].

### 2.2. Isolation of Human RBCs

Human blood from a healthy volunteer was collected into a Vacutainer tube containing heparin sulfate (Becton-Dickinson, Franklin Lakes, NJ). Whole blood was centrifuged at 250 × g at 4 °C for 5 minutes to collect RBCs. The RBCs were washed 3–4 times in PBS until the supernatant was clear. One hundred microliters of washed RBCs was added to 3.9 mL PBS to yield a 2.5% RBC suspension. This mixture was used for all RBC studies described here. Experiments involving fresh blood from human subjects were approved by the UMDNJ Institutional Review Board (IRB). All human subjects gave informed consent to participate.

### 2.3. Preparation of LtxA and LtxA-FITC

Leukotoxin (LtxA) was purified from culture supernatants of *A. actinomycetemcomitans* strain NJ4500 as previously described [24,25]. Briefly, culture supernatants were filtered and then ammonium sulfate precipitated to isolate protein. The ammonium sulfate pellet was then resuspended in LtxA buffer (20 mM Tris-HCl, pH 6.8, 250 mM NaCl, and 0.2 mM CaCl_2_) and passed over a Sephadex G-100 size exclusion column. Individual fractions were assayed for total protein content and purified LtxA. All toxin preparations were filtered through a 0.22 μm membrane prior to use. LtxA-FITC was prepared using the FITC (fluorescein 5-isothiocyanate) labeling kit (Thermo Scientific, Rockford, IL) as described by the manufacturer.

### 2.4. Ganglioside Blocking Assays

Purified bovine gangliosides (Sigma-Aldrich, St. Louis, MO) were diluted to the indicated concentrations in water and then filtered prior to use. Ganglioside (6 μL) was mixed with LtxA (15 μL; 0.2 mg/mL unless otherwise indicated) and incubated at room temperature for 20 minutes prior to adding to cells. The ganglioside-LtxA mixture was then added to cells (400 μL) and incubated at 37 °C for 24 hours unless otherwise indicated. 

### 2.5. Flow Cytometry

RBCs were stained with phycoerythrin (PE)-labeled anti-glycophorin (clone HIR2) antibody (CD235ab; Biolegend, San Diego, CA) by mixing 100 μL RBCs with 20 μL of a 1:10 dilution of the antibody. Cells were washed three times in PBS and then resuspended in 600 μl PBS. To assess LtxA-FITC binding to C6 glioma cells, LtxA-FITC (15 μL; 0.2 mg/mL) was pre-incubated with ganglioside (6 μL) for 15 minutes on ice and then the mixture was added to 0.1 mL C6 cells (10^6^ cells/mL), incubated for 30 minutes on ice, and then washed three times in PBS. Samples (at least 10,000 cells/run) were analyzed with a FACSCalibur instrument (BD Biosciences, Franklin Lakes, NJ) and data was analyzed using FlowJo software (Ashland, OR).

### 2.6. Cytotoxicity Assays

To determine the cytotoxic effect of LtxA on non-RBCs, 0.1 mL cells (~10^6^ cells/mL) were mixed with purified LtxA at various concentrations. The mixture was incubated at 37 °C, 5% CO_2_ for 24 hours unless otherwise noted. Cellular viability (ATP production) was then determined using the CellTiter-Glo luminescent cell viability assay (Promega, Madison, WI) according to the manufacturer’s instructions. Plates were read in a Synergy HT plate reader in the luminescence mode (Bio-Tek, Winooski, VT). For toxicity of RBCs, 0.1 mL cells were mixed with LtxA (8 μg/mL) and lysis was measured by detection of released hemoglobin. After 24-hour incubation, RBCs were removed by centrifugation and the absorbance of the supernatant at 450 nm was assayed on a Synergy HT plate reader. One hundred percent cell lysis was determined by resuspending RBCs in distilled water. 

### 2.7. Statistical Analysis

Data was analyzed using a Student’s t-test to test for no differences between ganglioside-treated and ganglioside-untreated samples. P values of <0.05 were considered significant.

## 3. Results

### 3.1. LtxA Does Not Use Glycophorin as a Receptor

**Figure 1:**
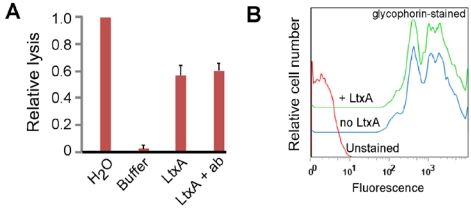
Effect of glycophorin and LtxA on RBCs. (a) RBCs were treated with water (100% lysis), LtxA buffer (background lysis), LtxA, or first pre-treated with anti-glycophorin antibody (ab) and then LtxA. Lysis was measured by detecting released hemoglobin in the supernatant at 450 nm. The data shown is representative of three independent experiments. (b) Flow cytometry of RBCs stained with anti-glycophorin antibody alone or after pre-treatment with LtxA. The shift in signal to the right represents cells that are stained with anti-glycophorin antibody. The data shown is representative of three independent experiments.

It was previously reported that *E. coli* α-hemolysin can use glycophorin, a sialoglycoprotein, on the surface of RBCs, as a receptor [23]. We asked if LtxA could also use glycophorin to cause lysis of RBCs. We first tested if anti-glycophorin antibody that reacts with both glycophorin A and B could block LtxA-mediated hemolysis. RBCs were pre-incubated in anti-glycophorin antibody for 30 minutes on ice, washed, and then mixed with LtxA. We found that anti-glycophorin antibody did not block hemolysis by LtxA (Figure 1A). Two different monoclonal antibody clones were tested in these studies yielding identical results (clones HIR2 and E3). In contrast, anti-LFA-1 antibody does block LtxA-mediated killing of WBCs [13,14]. We next asked if LtxA could block anti-glycophorin antibody from binding to RBCs. We performed flow cytometry to assay antibody binding to the surface of RBCs. Pre-incubation of RBCs with LtxA at 4 °C (a temperature that prevents LtxA from lysing cells) for 30 minutes reproducibly had no effect on anti-glycophorin antibody binding to RBCs (Figure 1B). Thus, these results suggest that LtxA does not use glycophorin as a RBC receptor.

### 3.2. Gangliosides Block LtxA-Mediated Hemolysis

**Figure 2:**
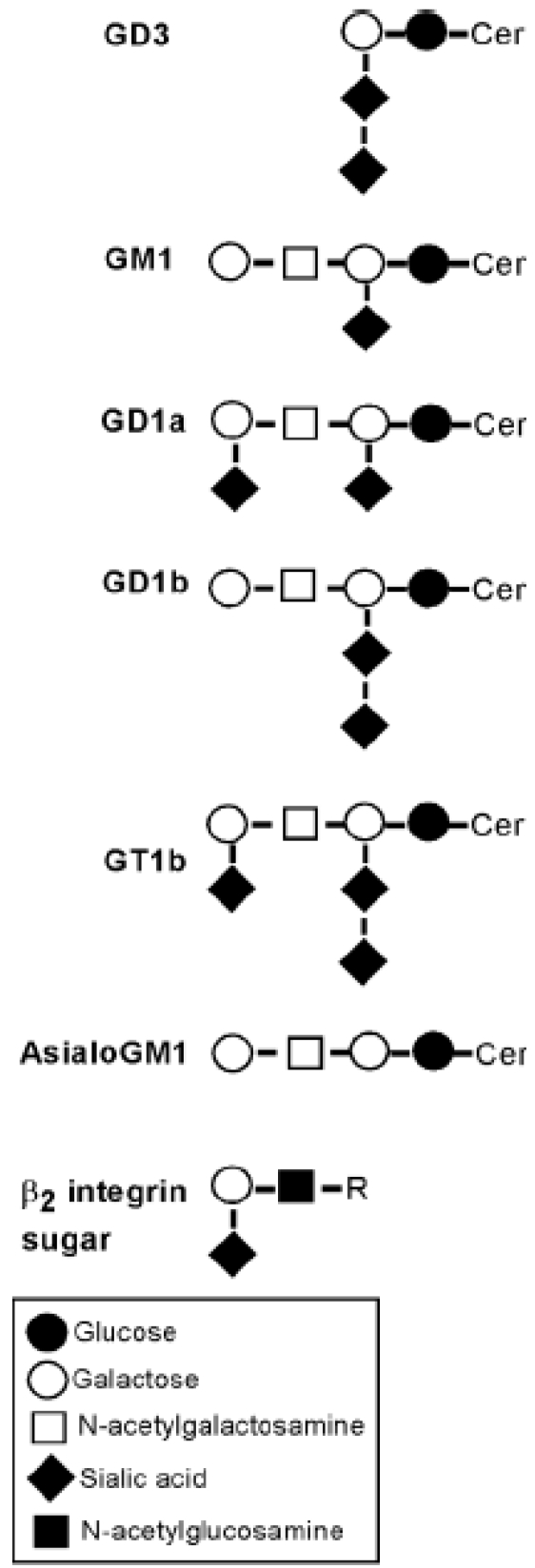
Structures of gangliosides and protein-linked carbohydrates. GD3, GM1, GD1a, GD1b, GT1b, and asialoGM1 are gangliosides. Cer, ceramide; R, complex-type sugar chain as described in [15].

**Figure 3:**
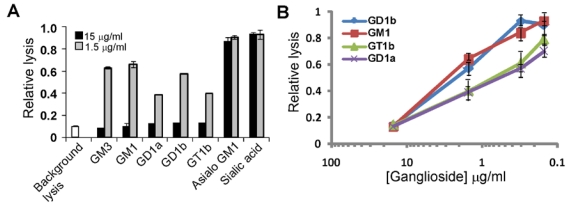
Gangliosides block LtxA-mediated lysis of RBCs. (A) LtxA (7.5 μg/mL) was pre-incubated with sugars (15 μg/mL or 1.5 μg/mL) noted on the x-axis for 20 minutes and then added to RBCs. RBCs were also treated with LtxA buffer to represent background lysis. Experiment was performed in triplicate and standard deviation error bars are shown. GM3, GM1, GD1a, GD1b, and GT1b all caused significant inhibition of LtxA-mediated lysis (P < 0.05). (B) Gangliosides block hemolysis by LtxA in a dose-dependent manner. Results are the average from three independent experiments. LtxA buffer caused relative background lysis of approximately 0.1. All values are relative to LtxA alone (set to 1.0).

We screened a panel of carbohydrates and carbohydrate-containing molecules to identify compounds that might block LtxA-mediated hemolysis. We found that only two of the compounds, gangliosides GM1 and GD1b, were able to block hemolysis nearly completely (Table 1). The screen was performed three independent times to confirm these results. Other bacterial toxins, including cholera toxin, *E. coli* heat-labile toxins, tetanus toxin, botulinum toxin, pertussis toxin, and shiga toxin, have been shown to use gangliosides as cellular receptors [26,27,28,29]. Gangliosides are glycosphingolipids found on the surfaces of many vertebrate cell types, especially RBCs and brain cells, and play a role in signaling and membrane protein regulation [30]. They consist of a lipid ceramide moiety attached to an oligosaccharide chain that contains at least one sialic acid residue (Figure 2). We next tested several purified gangliosides for their ability to block LtxA-mediated hemolysis. LtxA was pre-incubated in individual gangliosides for 20 minutes and then mixed with RBCs and incubated for 24 hours at 37 °C to allow hemolysis to occur. Figure 3A shows that five different gangliosides (GM3, GM1, GD1a, GD1b, and GT1b) were able to completely prevent LtxA-mediated hemolysis. The blocking effect was dose-dependent as shown in Figure 3B. In contrast, asialo GM1, lacking the sialic acid side group, was unable to fully block hemolysis. This result suggests that sialic acid is required for LtxA to interact with RBCs. Therefore, we also tested if sialic acid alone was sufficient to block hemolysis (Figure 3A). We found that sialic acid, even at high concentrations (15 μg/mL), was unable to completely prevent LtxA from lysing RBCs (Figure 3A). These results show that collectively, for LtxA recognition of RBCs, sialic acid is a necessary component of gangliosides, but alone is not sufficient to block lysis. To confirm that the gangliosides were binding to LtxA and not something on the surfaces of RBCs, we first pre-incubated RBCs with the gangliosides (GM3, GM1, GD1a, and GD1b). The RBCs were then washed, mixed with LtxA and incubated at 37 °C for 24 hours. We found that pre-incubation of RBCs with gangliosides did not block LtxA from lysing RBCs, indicating that the gangliosides are acting on LtxA (data not shown).

**Table 1:** Compounds that were screened for their ability to block LtxA-mediated hemolysis. + represents greater than 90% blocking of LtxA- mediated hemolysis; - represents less than 10% blocking of LtxA- mediated hemolysis.

Compound	Blocking
α-lactose	**-**
D (-) fructose	**-**
D (+) galactose	**-**
D-glucose	**-**
D-maltose	**-**
D-arabinose	**-**
D-ribose	**-**
Sucrose	**-**
D (+) mannose	**-**
D (+) xylose	**-**
N-acetylmuramic acid	**-**
Ganglioside GM1	**+**
Ganglioside GD1b	**+**

### 3.3. LtxA Binds to Gangliosides on Glioma Cells

**Figure 4:**
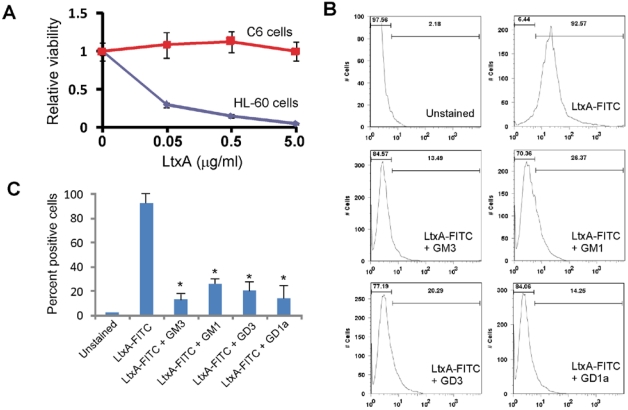
Interaction between LtxA-FITC and C6 glioma cells. (A) C6 or HL-60 cells were mixed with varying amounts of LtxA and then incubated for 24 hours. Viability was measured using the CellTiter-Glo viability assay. The experiment was performed in triplicate and standard deviation error bars are shown (P < 0.05). (B) Detection of LtxA-FITC binding to C6 cells using flow cytometry. Cells were stained with LtxA-FITC alone or LtxA-FITC that was pre-incubated (20 minutes) with the ganglioside noted. The left-most bar displays the percent of cells that are negative and the right-most bar represents percent of cells positive for staining with LtxA-FITC. The values on the x-axis represent fluorescence intensity. Results are representative of three experiments. (C) Histogram of the data shown in (B) from three experiments. * represents P < 0.05.

Studies with other bacterial toxins that bind gangliosides have employed glioma cell lines, such as rat glioma C6 cells, because they are enriched in gangliosides on their surfaces [27]. Because glioma cells should not be killed by LtxA, we could study binding of LtxA without the complications of downstream events such as apoptosis. We first confirmed that LtxA did not kill C6 glioma cells. After a 24-hour incubation with LtxA, the viability of C6 cells was not affected by any concentration of toxin tested in contrast to HL-60 cells (Figure 4A). To assay binding of LtxA to cells, we generated fluorescently-tagged LtxA (see Materials and Methods). Similar studies have been carried out using a FITC-labeled version of cholera toxin [31,32]. Modified LtxA (designated LtxA-FITC) was still active against HL-60 cells, indicating that covalent modification did not adversely affect activity of the toxin (data not shown). We next measured binding of the toxin to cells using flow cytometry (Figure 4B). LtxA-FITC stained C6 cells strongly (Figure 4B), but not K562 cells (data not shown), which lack LFA-1expression [14,19]. More than 90% of the cells stained with LtxA-FITC. However, pre-incubation of LtxA-FITC with gangliosides GM3, GM1, GD3, and GD1a decreased staining of C6 cells (Figure 4B and Figure 4C). Pre-incubation resulted in an approximately 80% decrease in staining per cell. These results were highly reproducible.

### 3.4. Gangliosides Inefficiently Block LtxA-Mediated Killing of WBCs

**Figure 5:**
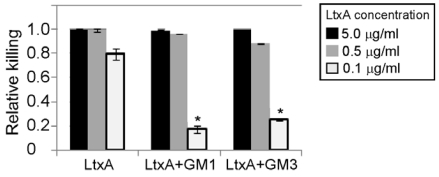
Gangliosides inefficiently block LtxA-mediated toxicity of WBCs. THP-1 cells were treated for three hours with LtxA alone or LtxA pre-incubated with ganglioside GM1 or GM3. The experiment was performed three times and the averages and standard deviations are shown. * represents P < 0.05.

LtxA is highly specific for human and Old World primate WBCs. We thus determined if gangliosides could also block LtxA-mediated killing of THP-1 WBCs. We found that, in contrast to lysis of RBCs, gangliosides GM1 and GM3 were not highly effective at blocking WBC cytotoxicity even though the ratio of GM1 molecules to LtxA molecules was 150:1 (Figure 5). Partial blocking was observed at lower doses of LtxA (~100 ng/mL; GM1 molecules to LtxA molecules ~7000:1); however, at higher concentrations of toxin, the gangliosides were largely ineffective at inhibiting LtxA-mediated toxicity. In addition, these experiments with THP-1 cells had to be reduced to three hours (RBC assays were performed for 24 hours); otherwise, LtxA overcame the inhibitory effects of the gangliosides after longer incubation times (data not shown). Thus, gangliosides can partially block killing of WBCs by LtxA only when the ratio of ganglioside to LtxA is high.

## 4. Discussion

We show here that RBC lysis by *A. actinomycetemcomitans* LtxA is efficiently blocked by soluble gangliosides. Gangliosides also abrogate binding of LtxA-FITC to ganglioside-rich glioma cells. Numerous bacterial toxins utilize gangliosides as cellular receptors on various cell types. These toxins are highly selective for specific gangliosides. For example, cholera toxin and *E. coli* heat-labile toxin bind to GM1, botulinum toxin to GT1b and GQ1b, and pertussis toxin to GD1a [26]. In contrast, based on the work presented here, LtxA does not show a preference for any specific ganglioside. To our knowledge, only one other bacterial toxin, *Clostridium perfringens* δ toxin, has been shown to interact with a ganglioside on RBCs, namely GM2 [33].

While gangliosides effectively prevented LtxA-mediated lysis of RBCs, they were much less effective at protecting WBCs from LtxA. LFA-1, the WBC receptor for LtxA, is a heavily glycosylated membrane protein [15]. Asada *et al.* [15] reported that ~60% of the oligosaccharide moieties that modify LFA-1 have the terminal structure: sialic acid→galactose→N-acetylglucosamine (Figure 2). Interestingly, this terminal sugar structure found on LFA-1 is strikingly similar to the gangliosides that block LtxA (Figure 2). In addition, Morova *et al.* [34] recently reported that RTX toxins can recognize β_2_ integrin receptors through their N-linked oligosaccharide chains and suggest that interaction between toxin and oligosaccharide represents the initial binding step. They showed that treatment of Jurkat T-cells with glycosidases rendered these cells more resistant to killing by RTX toxins, including LtxA. However, LtxA was still able to kill glycosidase-treated cells, but less efficiently than untreated cells. This result suggests that the deglycosylation was not complete or that LtxA can still interact with deglycosylated LFA-1, albeit less efficiently. WBCs have been reported to also express gangliosides on their surfaces [35,36]. However, cell lines that do not express LFA-1 (CD11a, CD18, or both) are completely resistant to LtxA-mediated toxicity [14,19], indicating that LtxA does not function through gangliosides on WBCs. This result is similar to the effect of LtxA on C6 glioma cells, which are recognized by LtxA but not killed. Thus, it appears that gangliosides do not act as potential functional receptors on cells except RBCs.

Based on several lines of evidence, we hypothesize that LtxA originally evolved as a toxin to target the immune system by interacting with LFA-1, but because of the similarities between the N-linked oligosaccharides of LFA-1 and gangliosides on the surface of RBCs, the toxin “gained” the ability to recognize and lyse RBCs. First, all of the sialidated gangliosides we tested were able to block LtxA-mediated hemolysis equally well with no apparent preference. All other bacterial toxins that interact with ganglioside receptors show strong preference for one or two gangliosides [26]. Second, gangliosides were only partially effective at blocking LtxA-mediated killing of WBCs (THP-1 cells) at low doses of LtxA and short incubation times. At high LtxA doses, blocking was not apparent. Third, when LtxA bound to C6 glioma cells via gangliosides, there was no subsequent toxicity or cellular changes, even at high doses after 24 hours. Interaction between other toxins and gangliosides always leads to noticeable downstream effects, especially, cell death. 

Interaction between RTX toxins and cellular receptors, such as LFA-1, leads to membrane disruption [37,38,39] and cellular signaling that ultimately results in cell death [40,41,42,43]. However, because RBCs lack LFA-1 and there is unlikely to be an intracellular signaling cascade activated by LtxA in RBCs, we suggest that interaction between LtxA and RBC gangliosides results in disruption of the membrane, which leads to cell lysis. Following interaction with LFA-1 on WBCs, LtxA is proposed to undergo significant conformational changes that result in membrane insertion of the toxin [39]. Thus, contact between LtxA and gangliosides may result in a similar change in conformation that allows LtxA to insert into and disrupt RBC membranes. Further biochemical studies will be required to test our hypothesis.

In conclusion, we demonstrate that gangliosides can block hemolysis by a toxin from an important oral pathogen. Our data and knowledge of other bacterial toxins suggest that gangliosides may act as a RBC receptor for LtxA. For individuals with HACEK-causing IE, the most common route of infection is through the oral cavity since the HACEK bacteria are part of the normal oro-pharyngeal flora. *A. actinomycetemcomitans* can be found on infected heart tissue where it exists as vegetative growths [9]. This indicates that *A. actinomycetemcomitans* has the ability to enter the blood stream, evade host immune defenses, colonize heart tissue, and persist in this environment. Furthermore, one of the clinical symptoms of IE is marked anemia, and hemolysis is considered to be an important mechanism leading to this anemia [44,45]. Thus, targeting of host RBCs by *A. actinomycetemcomitans* may be a crucial step in the pathogenic process and understanding this interaction could lead to novel therapeutic modalities. 
